# Detection and Characterization of *Staphylococcus aureus* and Methicillin-Resistant *S. aureus* in Foods Confiscated in EU Borders

**DOI:** 10.3389/fmicb.2017.01344

**Published:** 2017-07-21

**Authors:** David Rodríguez-Lázaro, Elena-Alexandra Oniciuc, Patricia G. García, David Gallego, Isabel Fernández-Natal, Marta Dominguez-Gil, José M. Eiros-Bouza, Martin Wagner, Anca I. Nicolau, Marta Hernández

**Affiliations:** ^1^Microbiology Division, Department of Biotechnology and Food Science, Faculty of Science, University of Burgos Burgos, Spain; ^2^Faculty of Food Science and Engineering, Dunarea de Jos University of Galati Galati, Romania; ^3^Laboratory of Molecular Biology and Microbiology, Instituto Tecnológico Agrario de Castilla y León Valladolid, Spain; ^4^Dependencia de Sanidad de Vizcaya, Delegación del Gobierno en el País Vasco Bilbao, Spain; ^5^Department of Clinical Microbiology, Complejo Asistencial Universitario de León León, Spain; ^6^Institute of Biomedicine, University of León León, Spain; ^7^Department of Clinical Microbiology, University Hospital Rio Hortega Valladolid, Spain; ^8^Institute for Milk Hygiene, Milk Technology and Food Science, University of Veterinary Medicine Vienna Vienna, Austria; ^9^Departamento de Ingeniería Agrícola y Forestal, Tecnología de los Alimentos, Escuela Técnica Superior de Ingenierías Agrarias, Universidad de Valladolid Palencia, Spain

**Keywords:** MRSA, food contamination, typing, illegal import, food safety, EU

## Abstract

The aim of the study was to evaluate the potential role of the illegal entry of food in UE in the Methicillin-resistant *S. aureus* (MRSA) spread. We studied the prevalence and characteristics of *Staphylococcus aureus* and MRSA isolated from foods of animal origin confiscated from passengers on flights from 45 non-EU countries from 2012 to 2015 by the Border Authorities at Bilbao International Airport (Spain) and Vienna International Airport (Austria), as well as foods from open markets close to EU land borders. Of 868 food samples tested (diverse meat samples including antelope, duck, guinea pig, pork, rodents, turkey, dairy products, and eggs), 136 (15.7%) were positive for *S. aureus* and 26 (3.0%) for MRSA. All MRSA strains were *mec*A-positive. The prevalence of *S. aureus*-positive dairy samples among food confiscated at Bilbao International Airport was 64.6%, and this airport also had the highest value (11.8%) for MRSA-positive samples. The predominant sequence type was ST5 (30.8%), followed by ST8, ST1649, ST1, and other lineages were found to a lesser extent (ST7, ST22, ST72, ST97, and ST398). Six isolates tested positive for *luk*-PVL genes (SCC*mec* IV subtypes IVc and IVe). Enterotoxin profiling revealed that 19 MRSA strains were enterotoxigenic, harboring one or more *se* genes. The MRSA isolates positive for *luk*-PVL genes were not enterotoxigenic, and none of the isolates tested positive for enterotoxin E. We found 14 resistance profiles, and more than 69% of the MRSA isolates were resistant to three or more types of antimicrobial agents. This finding reveals both the wide diversity of the antimicrobial resistance found in the strains and the capacity to resist not only to beta-lactam drugs. One MRSA strain showed unusual characteristics: it was oxacillin-susceptible, harbored SCC*mec* V, and was positive for *sed, seg*, and *sej* but negative for PVL virulence factors. This study shows the presence of enterotoxigenic HA-, CA-, and LA-MRSA in foods illegally entering the EU, and highlights illegal importation of food as route of enterotoxigenic MRSA spread. Uncontrolled entry of food stuffs into the EU can be a relevant neglected route of MRSA dissemination.

## Introduction

*Staphylococcus aureus* is a well-known opportunistic foodborne pathogen, and is involved in numerous nosocomial and community-associated (CA) outbreaks worldwide (Paterson et al., 2014). The widespread use of antibiotics, and particularly inappropriate use or overuse, has facilitated the emergence of pathogens resistant to antibiotics, such as methicillin-resistant *S. aureus* (MRSA). In addition to human clinical applications, antibiotics are also used in veterinary medicine and animal feeding, contributing to the substantial appearance of antibiotic-resistant strains (Valsangiacomo et al., 2000; Grema et al., 2015). The first reported nosocomial episode involving MRSA was in 1960, and MRSA has become an emergent pathogen (Bonten and Weinstein, 2016; Oniciuc et al., 2017), affecting patients in hospitals and people in community settings such as nursing homes and nurseries (Lo et al., 2007; Murphy et al., 2012; Blumental et al., 2013). MRSA has been found in livestock, linked to a jump from humans to animals (Voss et al., 2005; Price et al., 2012). The prevalence of livestock-associated (LA)-MRSA in farm animals is increasing and the resulting food products may become contaminated. This may be an important ecological niche, favoring the evolution of LA-MRSA (Yan et al., 2014; Verhegghe et al., 2016), and the apparently increasing prevalence can lead to human outbreaks (Grøntvedt et al., 2016).

Lineages other than LA-MRSA are found in food intended for human consumption. Contaminated food of animal origin may contribute to the prevalence of CA-MRSA (Ogata et al., 2012; Rodríguez-Lázaro et al., 2015) or hospital-acquired (HA)-MRSA (Pu et al., 2009; Weese et al., 2010). We demonstrated the presence of CA-MRSA in foods confiscated from passengers on international flights, mostly from Central and South America (Rodríguez-Lázaro et al., 2015). This has food safety implications: contamination with the CA-MRSA lineages isolated may have been due to incorrect food handling, and not zoonotic transmission.

Staphylococcal enterotoxins (SEs, *se* genes) have been detected in many foodstuff causing staphylococcal food poisoning, toxic shock, and allergic and autoimmune reactions (Gonano et al., 2009). Staphylococcal enterotoxins are classified into 23 different SEs and SE-like toxins (SELs) including the five major serological types SEA, SEB, SEC, SED, and SEE (Hennekinne et al., 2012; Carfora et al., 2015; Ono et al., 2015). However, little is known about the prevalence of MRSA in foods involved in international trade, or about their enterotoxin production. The role, if any, of foods illegally transported between different parts of the globe in MRSA epidemiology is unknown. We investigated whether the uncontrolled entry of foods into Europe through international flight passengers or from open markets at European Union (EU) borders is a potential route of transmission of enterotoxigenic antibiotic-resistant strains, particularly MRSA. Our study reveals the entry of MRSA by this route, and that the lineages involved showed enterotoxigenicity. Uncontrolled entry of foodstuffs into the EU can be a relevant neglected route of MRSA dissemination.

## Materials and Methods

### Food Sample Collection

This study was part of the EU-funded project PROMISE^1^ “*Protection of consumers by microbial risk mitigation through combating segregation of expertise.*” One of the PROMISE’s goals was to evaluate neglected risks for transmission of foodborne pathogens through exogenous routes of transmission (i.e., from non-UE countries) evaluating several UE international airports and borders (Wagner et al., 2015). A total of 868 animal-derived food items collected from August 2012 to July 2015 were tested for the presence of *S. aureus* and MRSA. The foods were confiscated from the luggage of passengers on flights from non-EU countries by the Border Authorities at Bilbao International Airport (Spain) (263 food products) and Vienna International Airport (Austria) (595 food products), or were collected from foods illegally introduced and sold in an open market closed to an EU border (the southeast part of Romania, on the border with Republic of Moldavia; Giurgiuleşti- Galaţi) (10 food products). Open markets at the Romanian borders are authorized to sell fresh vegetables, but not food of animal origin. Crossing the Romanian EU border with food is allowed just for low amounts of foods on distances that do not overpass 50 km (Romanian law 10/2010), but very often these foods are illegally sold in those open markets.

Food samples included 408 (47%) meat samples of diverse origin (including antelope, beef, chicken, duck, guinea pig, pork, rodents, and turkey), 447 (51.5%) milk and dairy products, 7 eggs (0.8%), and 6 fish and fish products (0.7%). The geographical origin of the food samples collected at the airports was wide: Africa (Côte d’Ivoire, Egypt, Ethiopia, Niger, South Africa, and Tunisia), America (Argentina, Bolivia, Brazil, Colombia, Cuba, Dominican Republic, Ecuador, Honduras, Mexico, Nicaragua, Panama, Paraguay, and Peru), Asia (Azerbaijan, China, India, Iran, Israel, Jordan, Kazakhstan, North Korea, Mongolia, Philippines, Qatar, South Korea, Thailand, Turkey, United Arab Emirates, and Vietnam), and non-EU Europe (Albania, Armenia, Bosnia and Herzegovina, Republic of Kosovo, Former Republic of Macedonia, Moldova, Republic of Serbia, Montenegro, Russia, and Ukraine).

### Detection and Isolation of *S. aureus*

*Staphylococcus aureus* was counted by the ISO 6888-2 method (ISO, 1999). Real-time PCR was used to confirm *S. aureus* isolates as previously described (Trnčíková et al., 2008). Positive colonies with the correct morphology on Baird Parker agar plates were taken for further typing tests (MRSA biotype, antibiotic resistance, and genetic characterization).

### Screening for the Presence of MRSA

*Staphylococcus aureus* isolates were tested for *mec*A and *mec*C by multiplex PCR as previously described (Stegger et al., 2012).

### Antibiotic Susceptibility Testing

Susceptibility to antimicrobials was determined by a microdilution method, applying the recommendations and minimum inhibitory concentration (MIC) breakpoints of the European Committee on Antimicrobial Susceptibility Testing (EUCAST) guidelines 2015 (EUCAST, 2015). Susceptibility to 20 antimicrobial agents was tested: penicillin (PEN), oxacillin (OXA), amoxicillin/clavulanate (AMC), daptomycin (DAP), erythromycin (ERY), clindamycin (CLI), teicoplanin (TEC), vancomycin (VAN), ciprofloxacin (CIP), levofloxacin (LVX), amikacin (AMK), gentamicin (GEN), tobramycin (TOB), mupirocin (MUP), rifampicin (RIF), tetracycline (TET), fusidic acid (FUS), fosfomycin (FOF), linezolid (LZD) and trimethoprim sulfamethoxazole (SXT).

### Characterization of the Genetic Background

All MRSA isolates were subjected to Pulsed Field Gel Electrophoresis (PFGE) (McDougal et al., 2003), Multi Locus Sequence Typing (MLST) (Enright et al., 2000) and typing and subtyping of the SCCmec (staphylococcal cassette chromosome *mec*) element (Kondo et al., 2007; Milheiriço et al., 2007). PFGE patterns were analyzed with Bionumerics software v6.6 (Applied-Maths NV, Sint-Martens-Latem, Belgium), and dendrograms were constructed using the Dice similarity coefficient and the unweighted pair group mathematical average (UPGMA) clustering algorithm with 1% tolerance and optimization values. The allelic profiles obtained by MLST were assigned by comparison with the *S. aureus* MLST database hosted at Saureus.mlst.net (2016). Information about MRSA strains was submitted to that database. Bionumerics software v6.6 was used for allelic profile-based comparisons applying a minimum spanning tree (MST) to define the relationships among MRSA strains at the micro evolutionary level. A sequence type (ST) number was attributed to each distinct combination of alleles at the seven genes (Ragon et al., 2008).

### Detection of Panton-Valentine Leukocidin Virulence Factors

All MRSA strains were tested for the PVL genes (*lukS*-PV and *lukF*-PV) by conventional PCR as described (Lina et al., 1999). Reference strain ATCC 49775 was used as a positive control.

### Enterotoxin Profiling

Methicillin-resistant *S. aureus* isolates were tested by a multiplex PCR targeting *sea, seb, sec, sed, see, seg, seh, sei, sej* genes as described by Gonano et al. (2009).

## Results

### MRSA in Food Samples Confiscated by Border Authorities

**FIGURE 1:**
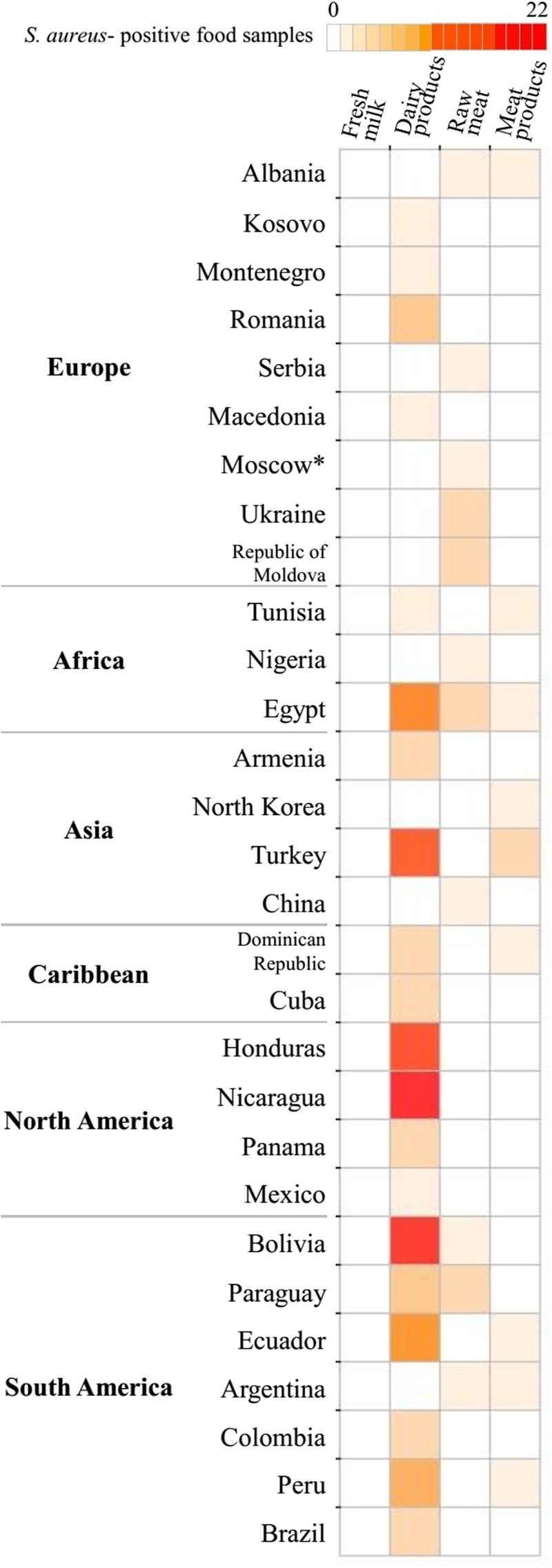
Heat mapof the origin and distribution of *Staphylococcus aureus*-positive food samples.

**FIGURE 2:**
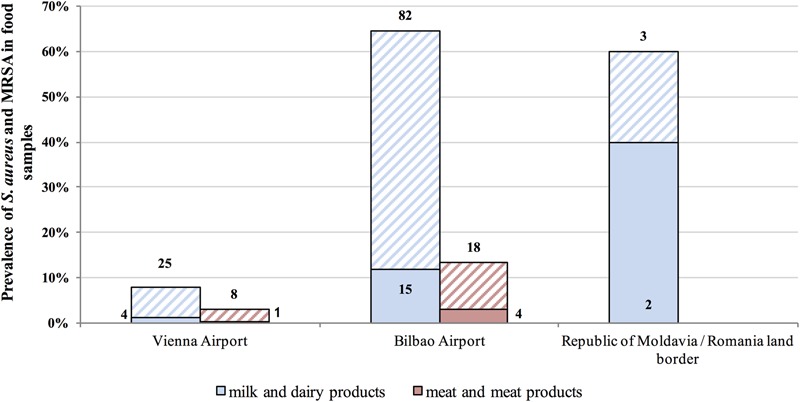
Prevalence (%) of *S. aureus* in food samples. Columns with stripes represent *S. aureus*-positive isolates and solid columns represent methicillin-resistant *S. aureus* (MRSA)-positive samples.

**FIGURE 3:**
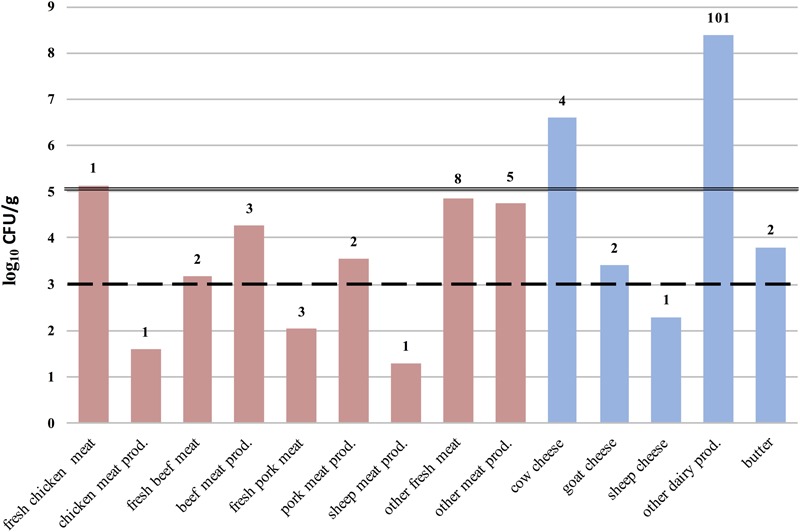
*Staphylococcus aureus* counts (log_10_CFU/g) *per* food category, type and origin. The number of food samples analyzed *per* food type are displayed above each column. Lines passing through the columns represent: (- -) the maximum (M) acceptable value in the microbiological criteria for raw milk intended for processing and in powdered milk; and ( – ) the M acceptable value for cheeses made from raw milk, according to Anonymous (2005). The International Commission on Microbiological Specifications for Foods (ICMSF) recommends an M acceptable value of 10^3^ CFU/g for cooked meat and poultry products (ICMSF, 2011).

Microbiological tests revealed *S. aureus* in 15.7% of confiscated food items and MRSA in 3.0% (26/868) (**Figures 1, 2**). The highest prevalence of *S. aureus* we observed was 64.6% in dairy samples confiscated at Bilbao Airport, and the highest value for MRSA-positive samples was 11.8% (**Figure 2**). The mean *S. aureus* count overall was 2.9 × 10^6^ CFU/g, with a minimum value of 1.0 × 10^1^ CFU/g in raw pork meat confiscated at Bilbao Airport from a passenger flying from Moscow, and a maximum value of 2.45 × 10^8^ CFU/g in cheese confiscated at Vienna Airport from a passenger flying from Turkey (**Figure 3**).

The MRSA strains were isolated from 21 milk and dairy products (cow, sheep or goat milk and cheese, either fresh, brined or with spices), and five meat and meat products (raw and cooked meat). The MRSA-positive foods confiscated at Bilbao Airport were from flights from Nigeria (1), Egypt (2), Republic of Honduras (1), China (1), Nicaragua (5), Bolivia (4), Ecuador (1), Peru (2), Columbia (1), and Republic of Serbia (1). Those at Vienna Airport, were from flights from Egypt (3) and Turkey (2). Two MRSA-positive food samples were from the Republic of Moldova and were confiscated at the Romanian border.

### Antibiotic Profile of the MRSA Isolates

Antibiotic susceptibility testing identified 14 resistance profiles (**Table 1**). Sixteen of the 49 MRSA strains were multiresistant.

**Table 1:** Antibiotic resistance profiles of methicillin-resistant *S. aureus* (MRSA) strains isolated from food confiscated from passengers of non-EU-flights or at a land border, 2012–2015.

Resistance profile	Antibiotics^a^	Number of strains	Percentage (%)
RP0	β-lactams	6	22.2
RP1	PEN, TET, ERY	5	18.5
RP2	PEN, ERY	3	11.1
RP3	PEN, FUS, TET, TOB, GEN	2	7.4
RP4	PEN, TET, TOB	2	7.4
RP5	PEN, TET, SXT	1	3.7
RP6	PEN, TET, FUS	1	3.7
RP7	PEN, FOF	1	3.7
RP8	PEN, LVX	1	3.7
RP9	PEN, LVX, SXT	1	3.7
RP10	PEN, LVX, FOF, RIF	1	3.7
RP11	PEN, TET, ERY, CLI	1	3.7
RP12	[PEN]^b^, TET, ERY, [OXA]^b^	1	3.7
RP13	PEN, TET, CIP, LVX, ERY, CLI	1	3.7

*PEN, penicillin; FOF, fosfomycin; TET, tetracycline; SXT, trimethoprim sulfamethoxazole; FUS, fusidic acid; ERY, erythromycin; LVX, levofloxacin; TOB, tobramycin; RIF, rifampicin; GEN, gentamicin; CLI, clindamycin; CIP, ciprofloxacin; MUP, mupirocin. ^a^The minimum inhibitory concentration (MIC) break points used were those in the EUCAST (2015) guidelines. ^b^Parentheses indicates susceptibility.*

### Genetic Characterization of MRSA Isolates

All the MRSA isolates harbored the *mec*A gene and none harbored the *mec*C gene. More than 75% of the isolates were SCC*mec* type IV: 48.9% were IVc and IVe, 22.4% IVa, and 4.1% IVh, and 24.5% were SCC*mec* type V. SCC*mec* typing of three isolates was not possible: the multiplex PCR-2, which types the *mec*A complex class, amplified a 804 bp DNA fragment, consistent with type C, but the multiplex PCR-1 targeting the *ccr* gene complex did not give an amplification product. Similarly, for another isolate the multiplex PCR-1 amplified a 937 bp DNA fragment consistent with *ccr* type 2 (A2B2), but the multiplex PCR-2 did not give an amplification product. In addition, six isolates tested positive for *luk*-PVL genes (all of them were SCC*mec* IV subtypes IVc and IVe).

Most of the MRSA isolates were positive for the enterotoxin genes A, B, C, D, G, H, I, J, but none were positive for enterotoxin E. Nineteen of the 26 MRSA strains (73%) tested positive for one or more *se* genes (**Table 2**); four strains (15.4%) harbored only one kind of *se* gene, and 15 strains (57.6%) more than one type of *se* gene (six strains carried *seg/sei* genes). None of the MRSA isolates positive for *luk*-PVL genes were enterotoxigenic.

**Table 2:** Enterotoxin profiles of MRSA strains.

Type of *se* gene	Milk and dairy products	Meat and meat products
*se*-negative	5 (19.2)^a^	2 (7.7)
*se*-positive	16 (61.6)	3 (11.5)
*sea*	1 (3.85)	–
*seg*	1 (3.85)	–
*seh*	2 (7.7)	–
*sea/seb*	3 (11.5)	1 (3.8)
*sea/seh*	1 (3.85)	–
*seg/sei*	4 (15.4)	2 (7.7)
*sec/seg/sei*	1 (3.85)	–
*sed/seg/sej*	1 (3.85)	–
*sed/seg/sei/sej*	2 (7.7)	–

*^a^Number and percentages in parenthesis of MRSA strains positive for enterotoxin-producing genes.*

**FIGURE 4:**
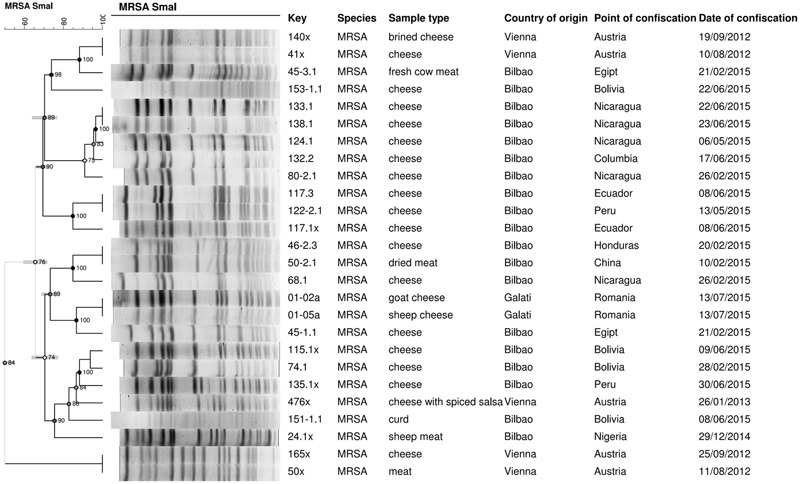
Genetic relationships among the *Sma*I Pulsed Field Gel Electrophoresis (PFGE) profiles of the 26 MRSA isolates. MRSA isolates were obtained from the 136 for *S. aureus*-positive food samples, confiscated from people traveling into the European Union (EU), from August 2012 to July 2015. The dendrogram was generated from Dice similarity coefficients with the unweighted pair group mathematical average (UPGMA) clustering algorithm with 1% in the tolerance and optimization values. The scale indicates similarity values.

Pulsed Field Gel Electrophoresis patterns and ST types were determined. *Sma*I-PFGE provided a fingerprint pattern consisting of 13–17 DNA fragments of 20–670 Kbp, approximately (**Figure 4**). Two isolates were not typeable by *Sma*I-PFGE suggesting they may be ST398, a lineage with an unusual resistance to digestion by *Sma*I (Chung et al., 2000). MLST identified nine ST, with eight MRSA strains (30.8%) being ST5. These eight ST5 isolates were of related genotypes: three showed the same fingerprint pattern, harboring SCC*mec* type V, and five, SCC*mec* type IV. The eight strains were isolated from food samples not confiscated on the same date, or at the same airport; they were cheese (*n* = 7) from Nicaragua, Columbia, Egypt, and Turkey and fresh beef meat (*n* = 1) from Egypt.

**FIGURE 5:**
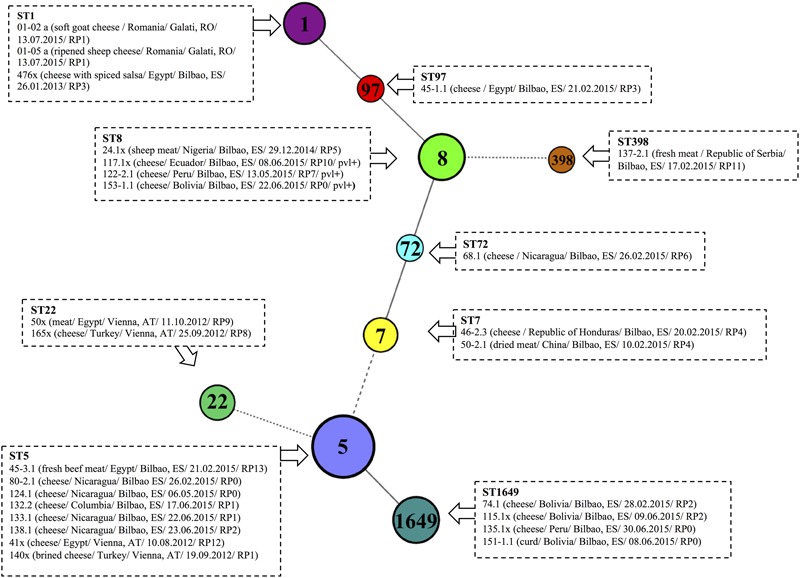
Multi Locus Sequence Typing (MLST) of 26 MRSA strains isolated from food illegally imported into the EU. The isolated were clustered according to seven housekeeping genes using a minimum spanning tree (MST). A different randomized color was attributed to each sequence type (ST). MRSA strains belonging to the same ST are displayed surrounded by dotted boxes. Information referring to sample type/country of origin/confiscation point/date of confiscation: DD.MM.YYYY/resistance profile is given in brackets for each MRSA strain, following the strain code. Abbreviations used: AT, Austria; ES, Spain; RO, Romania.

The other MRSA strains were ST1649, ST8, ST1, ST22, ST72, ST97, and ST398 (**Figure 5**). ST1649 (15.4%) and ST8 (15.4%) were the most prevalent (15.4% each), and the three ST8 carried PVL genes. ST1, ST22, ST72, and ST97 were found in different hard and semi-hard cheeses. ST398 is a livestock clone and was found in fresh meat confiscated from a passenger traveling from the Republic of Serbia to Bilbao Airport.

An oxacillin-susceptible *mec*A-positive *S. aureus* (OS-MRSA) strain was isolated from cheese confiscated from a passenger flying from Turkey. To our knowledge, this is the first time that an OS-MRSA has been isolated from processed food, and the first time that an OS-MRSA has been isolated from food in Europe. This might indicate that OS-MRSA may be circulating in Europe and may enter the EU via the illegal import of food.

## Discussion

We document *S. aureus* and MRSA in food confiscated to international flight travelers in UE airports or illegally sold foods in open markets at EU borders. Approximately one in six of the foods tested (136 of 868; 15.7%) were positive for *S. aureus*, and 3.0 % were positive for MRSA (26 of 868). The confiscated foods in which *S. aureus* counts exceeded international microbiological recommendations and EU regulations (Anonymous, 2005) were of very diverse geographical origins, including South and Central America, Europe, Africa, and Asia (**Figures 1, 2**). This is a serious public health concern because *S. aureus* can produce enterotoxins that can be present in food products. Our results agree with previous reports of the prevalence of *S. aureus* contamination (13–60%) of food products of animal origin (Crago et al., 2012; Rodríguez-Lázaro et al., 2015; Sun et al., 2015; Ciolacu et al., 2016). Various confiscated cheeses, including soft, semi hard, or hard had counts of up to 3.17 log_10_ above the limit established by Anonymous (2005). The high *S. aureus* counts may be in part the consequence of improper storage and poor hygiene according to the nature of surfaces and number of people handling the food (Chen et al., 2001). There may also be two way bacterial transfer between clothes and food preparation surfaces (Bloomfield et al., 2011). Twenty-six of the *S. aureus* strains isolated were MRSA (3.0%) of which 19 were enterotoxigenic. Under inappropriate food transportation conditions, heat-stable enterotoxins could emerge leading to gastroenteritis outbreaks. Many of the MRSA strains were positive for one or more *se* genes, consistent with previous studies (Pereira et al., 2009; Carfora et al., 2015). Contaminated milk and dairy products are major vectors of enterotoxins but, the link between the source of food contamination and transfer of antibiotic resistance determinants remains unclear; there are few reports describing the presence and possible origin of MRSA in foods (Ortega et al., 2010). However, there has been one case of community-acquired foodborne illness caused by SEC-producing MRSA (Jones et al., 2002) in the United States, and production of SE types SEB, SEC, SED, and SEE in two MRSA strains of milk origin from Minnesota farms (Haran et al., 2012).

Little is known about the prevalence of MRSA in food (Oniciuc et al., 2017), and particularly on in foods entering the EU. Our research group has performed several studies in airports and at borders (Oniciuc et al., 2015; Rodríguez-Lázaro et al., 2015) and found *S. aureus* and MRSA prevalence values of 8–33.9% and 0.5–3.1%, respectively. The values we report here are similar: 15.7 and 3.0% for *S. aureus* and MRSA, respectively. The number, the type of food sample, the geographical origin and the sampling place (Vienna International Airport was included in this study) are larger than in previous studies, but no significant differences in the overall percentages were observed. Nevertheless, 19 of 263 food items (7.2%) were MRSA positive at Bilbao Airport but only 7 of 595 (0.8%) at Vienna Airport. This significant difference could be a consequence of the geographical origins of the flights: most of the MRSA-positive foods at the Bilbao Airport were from Central and South America (14 of 19; 73.7%).

The MRSA lineages found included ST5, ST8, ST1649, ST1 and other lineages represented in a lesser extent (ST7, ST22, ST72, ST97, and ST398) (**Figure 5**). The ST most frequently isolated was ST5 (30.8%). This ST is believed to be the result of a species jump followed by adaption to the new host (Lowder et al., 2009). ST5 has mostly been found among poultry isolates (Lowder et al., 2009), but in our study was mostly isolated from dairy products. The ST5 lineage is a major component of hospital- and community-associated MRSA and MSSA worldwide (Miko et al., 2013). Other MRSA strains identified in our study were ST8-MRSA-IV/V and ST1649-MRSA-IV, which are successful of CA-MRSA clones. The presence of PVL genes and different antimicrobial susceptibility patterns in ST8-MRSA are causes of concern; it is not clear whether human handlers played any role in the preliminary post slaughter process.

The LA-MRSA ST398 is widespread in Europe (Oniciuc et al., 2017). It emerged rapidly in the Netherlands and now accounts for 20% of all human MRSA cases and for 42% of newly detected MRSA, indicating that animals are important reservoirs for human MRSA infection (Kadariya et al., 2014). There have already been outbreaks due to LA-MRSA ST398 (Wulf et al., 2008; Verkade et al., 2012). However, LA-MRSA ST398 was found only in one dairy product from Egypt in our study; this isolate harbors the *luk*-PVL genes but not enterotoxin-producing genes, in accordance with previous studies (Argudín et al., 2011). We isolated one OS-MRSA strain from a cheese from Turkey at Vienna airport: this MRSA variant is important because it may be misidentified as MSSA (Ariza-Miguel et al., 2015), such that β-lactam antibiotics may be used for treatment resulting in the potential development of highly resistant MRSA.

Many of our MRSA isolates were resistant to three or more antimicrobial agents (**Table 1**). This relatively high prevalence of these strains and their antimicrobial resistance profiles reveals the potential public health problems associated with illegal import of food in passenger luggage. Multiresistant strains are being distributed worldwide by air travel, and indeed undoubtedly by the various other forms travel across borders. United States custom officers ask all passengers from outside the United States to fill a form about contact with animals (Category A referring to zoonotic disease transmissible through animal contact) and any animal-origin food product potentially infected with zoonotic agents (Category B) (Noordhuizen et al., 2013). The EU forbids the importation of personal foodstuff of animal origin including meat, milk, and derived foodstuffs, from non-EU-countries (other than the Faeroe Islands, Greenland, and Iceland) (Anonymous, 2009). The EU has issued several regulations for the import of animals and food products of animal origin, but these regulations generally refer to commercial trade and bulk food products (Anonymous, 2007, 2009). This disregard of the risk associated with illegally transported foodstuffs combined with insufficient border controls could lead to foodborne outbreaks (Noordhuizen et al., 2013). The prevalence of MRSA in food depends on, among other factors, origin and country of provenance (Rodríguez-Lázaro et al., 2015; Oniciuc et al., 2015). Foodborne outbreaks of MRSA infection have been reported (Jones et al., 2002), confirming that food serves as a transmission pathway for MRSA (Oniciuc et al., 2017).

## Conclusion

This study shows the presence of enterotoxigenic HA-, CA-, and LA-MRSA in foods confiscated from passengers on flights entering the EU and in food illegally introduced and sold at EU borders. There is therefore a risk of foodborne transmission of MRSA, and illegal entry of food is a possible route for enterotoxigenic MRSA transmission and spread. Efficient control measures (such as the increase of the *in situ* examination and testing for the presence of uncontrolled entry of foods at EU borders) are required to avoid transmission of antimicrobial-resistant strains to humans by the consumption of such foods. Travelers should be made aware of the dangers, to promote acceptation of prohibition of food transport and thereby reduce the risk of spreading of foodborne pathogens.

## Author Contributions

DR-L designed, supervised the experiments, analyzed the results, revised the first draft, and prepare the last draft of the manuscript. E-AO performed part of the experiments, analyst the data and prepare the first draft of the manuscript. PG, IF-N, DG, and MD-G performed part of the experiments. JE-B performed part of the experiments and revised the different version of the manuscript. MW and AIN collaborated in the design of the experiment and revised the different version of the manuscript. MH collaborated in the design of the experiment, in the supervision of the experiments, and in the analysis of the results and revised the different versions of the manuscript.

## Conflict of Interest Statement

The authors declare that the research was conducted in the absence of any commercial or financial relationships that could be construed as a potential conflict of interest.

## Footnotes

1 www.promise-net.eu
